# Characterization of the complete mitochondrial genome of *Pseudorhaetus sinicus* Boileau, 1899 (Coleoptera: Lucanidae)

**DOI:** 10.1080/23802359.2021.1997656

**Published:** 2021-11-16

**Authors:** Zhibing Zhao, Jie Wang, Long Wu, Yu Bai, Chun Wang, Guiling Qi, Can Li, Yu Cao

**Affiliations:** a Guizhou Provincial Key Laboratory for Rare Animal and Economic Insect of the Mountainous Region, Guizhou Provincial Engineering Research Center for Biological Resources Protection and Efficient Utilization of the Mountainous Region, Guiyang University, Guiyang, China; b College of The Environment & Ecology, Museum of Biology, Xiamen University, Xiamen, China

**Keywords:** *Pseudorhaetus sinicus*, Lucanidae, mitochondrial genome, stag beetle, mitogenome

## Abstract

*Pseudorhaetus sinicus* is a stag beetle common to China and Vietnam, but whose distribution is limited within China. Little is known about the molecular biological characteristics of this species, so we characterized its complete mitochondrial genome (GenBank accession number MZ504793.1). The mitogenome consists of a circular DNA molecule of 18,126 bp, with 67.693% AT content. It contains 13 protein-coding genes (PCGs), 22 tRNA genes, and two rRNA genes. The PCGs have typical ATN (Met) start codons and TAN stop codons. Phylogenetic analysis suggests that *P*. *sinicus* is closely related to *Prosopocoilus confucius*. This newly described mitochondrial genome provides a valuable resource for the phylogenetic analysis of Lucanidae beetles.

*Pseudorhaetus sinicus*, which belongs to order Coleoptera, family Lucanidae, is distributed mainly in Vietnam and China. It is commonly found in Fujian, Zhejiang, Jiangxi, and Guizhou Provinces, China. The partial mitochondrial genome (KP987575.1) of *P. sinicus* from Daming Mountain in Guangxi Province, China, has previously been sequenced (Wu et al. 2016). Here, we have characterized the complete mitogenome of *P. sinicus* from Yong’an City, Fujian Province, China, to better understand the molecular evolution and taxonomic classification of *P. sinicus*.

A specimen of adult *P*. *sinicus* was collected in Longtou Valley (117.36517°N, 25.94136°E), Fujian Tianbaoyan National Nature Reserve, Yong’an City, Sanming City, China, on 24 September 2020, and deposited by Yu Cao (Email: yucaosuccess@126.com) in the animal specimen room of Guiyang University (specimen accession number: GYU-20200924-001). Total genomic DNA was isolated using an Aidlab Genomic DNA Extraction Kit (Aidlab Co., Beijing, China). Universal primers were designed (Supplementary Table 1) to match generally conserved regions to amplify short fragments from 12S and 16S rRNA, *cox1*, *cox2*, *nad1*, *nad2*, and *nad5*. PCR products were cloned into a pMD18-T vector (Takara Bio, Kusatsu, Japan) and then sequenced, or sequenced directly by the dideoxy nucleotide procedure, using an ABI 3730 automatic sequencer (Applied Biosystems, Foster City, CA). Thirty-six short sequences were obtained which range in length from 253 bp to 1081 bp.

The complete mitogenome of *P*. *sinicus* (GenBank accession number: MZ504793.1) was assembled manually. It is a circular DNA molecule that is 18,126 bp long (nucleotide composition: 36.2% A, 31.5% T, 21.2% C, and 11.2% G; 67.7% AT content). Using Perna and Kocher’s formula (Perna and Kocher 1995), the AT- and GC-skews of the major strand of the mitogenome were calculated to be 0.070 and −0.309, respectively. The AT-rich region of the mitogenome is 3549 bp long with 70.4% AT content, and is located between the genes that encode small regulatory RNA (srRNA) and tRNA-Ile.

The *P*. *sinicus* mitogenome contains 13 protein-coding genes (PCGs), 22 tRNA genes, and two rRNA genes, which were annotated using the MITOS web server (http://mitos.bioinf.uni-leipzig.de/) (Bernt et al. 2013). The transcriptional start and stop sites of the PCGs were manually corrected using *P. sinicus* (KP987575.1) from Daming Mountain, *Tenebrio obscurus* (Bai et al. 2018), *Zophobas atratus* (Bai et al. 2019), and *Blaps rhynchoptera* (Yang et al. 2019) genomic sequences as references. All 13 PCGs had typical ATN (Met) start codons. Three genes (*atp6*, *nad3*, and *nad1*) had ATA start codons, one (*nad2*) had an ATC start codon, five (*cox3*, *nad4*, *nad4l*, *nad6*, and *cob*) had ATG start codons, and four (*cox1*, *cox2*, *atp8*, and *nad5*) had ATT start codons. All 13 PCGs had typical TAN stop codons. Four genes (*atp8*, *atp6*, *nad4l*, and *nad6*) had TAA stop codons, four (*nad2*, *nad3*, *nad1*, and *cob*) had TAG stop codons, and five (*cox1*, *cox2*, *cox3*, *nad4*, and *nad5*) had incomplete stop codons that were completed by the addition of A nucleotides at the 3′ ends of the encoded mRNAs. The 22 tRNA-encoding genes were interspersed throughout the coding region and ranged from 61 bp (tRNA-Cys) to 71 bp (tRNA-Lys) long. The genes encoding large rRNA and srRNA were 1269 bp and 760 bp long, respectively.

To validate the phylogenetic position of *P*. *sinicus*, its mitochondrial PCGs and those of 16 other species in class Insecta were used to construct a maximum-likelihood phylogenetic tree with 1000 replicates using MEGA X software (Kumar et al. 2018) (Figure 1). The mitogenomes of *T. obscurus* (Bai et al. 2018) and *Z. atratus* (Bai et al. 2019) were selected as the outgroup. *P*. *sinicus* is closely related to *Prosopocoilus confucius* (Lin et al. 2017) and our tree is consistent with this. Overall, this study provides insights into the mitogenome of *P*. *sinicus*, and also provides essential genetic and molecular data for further phylogenetic and evolutionary analyses of family Lucanidae.

**Figure 1.**
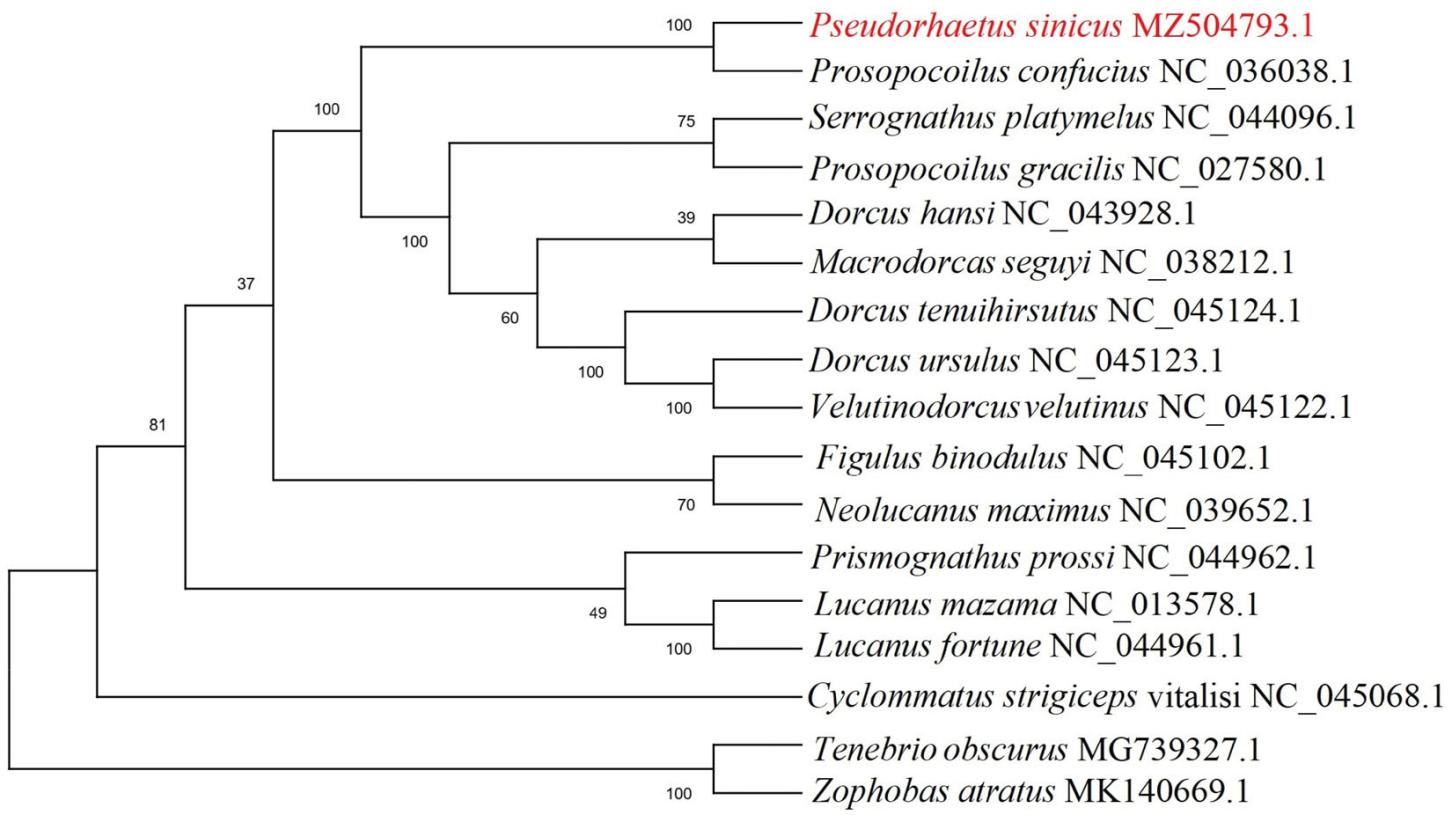
Maximum-likelihood phylogenetic tree of *Pseudorhaetus sinicus* and 16 other species in class Insecta based on the sequences of 13 protein-coding regions in their mitogenomes.

## Data Availability

The genome sequence data that support the findings of this study are openly available in GenBank at https://www.ncbi.nlm.nih.gov under accession no. MZ504793.1.
